# Supplementary material for: Resolving the full spectrum of human genome variation using Linked-Reads
Source: Genome Res. 2019 Apr;29(4):635–45. doi: 10.1101/gr.234443.118 (PMC6442396; doi:10.1101/gr.234443.118)
Supplement: Supplemental Material [file supp_gr.234443.118_Supplemental_longranger-2.2.zip › longranger-2.2.tar.bz2!/lr2.2-public/lib/stan/cmdstan/stan/doc/api/html/var__stack_8cpp.html]

Stan: src/stan/agrad/rev/var\_stack.cpp File Reference


|  |  |
| --- | --- |
|  | Stan  2.5.0  probability, sampling & optimization |


- Main Page
- Related Pages
- Namespaces
- Classes
- Files

- File List
- File Members

All Classes Namespaces Files Functions Variables Typedefs Enumerations Enumerator Friends Macros Pages

- src
- stan
- agrad
- rev

Namespaces |
Macros |
Variables

var\_stack.cpp File Reference

`#include <stan/agrad/rev/var_stack.hpp>`

Go to the source code of this file.

|  |  |
| --- | --- |
| Namespaces | |
|  | stan |
|  | Probability, optimization and sampling library. |
|  | |
|  | stan::agrad |
|  | Function gradients via reverse-mode automatic differentiation. |
|  | |

|  |  |
| --- | --- |
| Macros | |
| #define | \_\_STAN\_\_AGRAD\_\_REV\_\_VAR\_STACK\_CPP\_\_ |
|  | |

|  |  |
| --- | --- |
| Variables | |
| std::vector< chainable \* > | stan::agrad::var\_stack\_ |
|  | |
| std::vector< chainable \* > | stan::agrad::var\_nochain\_stack\_ |
|  | |
| std::vector< chainable\_alloc \* > | stan::agrad::var\_alloc\_stack\_ |
|  | |
| memory::stack\_alloc | stan::agrad::memalloc\_ |
|  | |
| std::vector< size\_t > | stan::agrad::nested\_var\_stack\_sizes\_ |
|  | |
| std::vector< size\_t > | stan::agrad::nested\_var\_nochain\_stack\_sizes\_ |
|  | |
| std::vector< size\_t > | stan::agrad::nested\_var\_alloc\_stack\_starts\_ |
|  | |

## Macro Definition Documentation

#define \_\_STAN\_\_AGRAD\_\_REV\_\_VAR\_STACK\_CPP\_\_

Definition at line 2 of file var\_stack.cpp.


---

[ Stan Home Page ]

*© 2011–2014,
Stan Development Team.*
